# Proactive risk assessment and nursing risk management in chemotherapy drugs: FMECA methodology results

**DOI:** 10.1002/jhrm.70021

**Published:** 2026-02-25

**Authors:** Gennaro Laus, Giuseppe Bertozzi, Aldo Di Fazio, Federico Ruta, Tommaso Cassano, Sipontina Rita Zerulo

**Affiliations:** ^1^ IRCCS CROB Rionero in Vulture Italy; ^2^ CIS of Legal Medicine of Basilicata Potenza Italy; ^3^ General Direction ASL BAT Barletta Italy; ^4^ Department of Medical and Surgical Sciences University of Foggia Foggia Italy; ^5^ University of Foggia Foggia Italy

## Abstract

The study aims to proactively analyze the chemotherapy drug management process, focusing on the nursing role: identifying error modes and proposing improvement interventions. A quality improvement project was conducted at an Onco‐Hematological Department, applying a systematic and prospective approach to evaluate the critical phases of the chemotherapy drug process. A risk analysis was conducted using Failure Mode, Effects, and Criticality Analysis (FMECA). A multidisciplinary team of healthcare professionals with experience in the sector mapped the process, identified potential failure modes, calculated the Risk Priority Numbers (RPNs), and implemented corrective actions. The RPNs were re‐evaluated after three months to measure intervention effectiveness. The analysis identified eight error modes, two of which were classified as high risk: contamination (initial RPN 27) and inaccurate dosing (initial RPN 24). After the implementation of improvement actions, such as specific training and formalized double checking, the RPNs were reduced to 4 and 3, respectively, with an improvement greater than 85%. The application of FMECA has allowed for effective identification and mitigation of the main risks associated with chemotherapeutic drug management, contributing to improve patient safety and nurse perceived quality of performance, through a model replicable in other healthcare settings.

## INTRODUCTION

Adverse drug events are unexpected incidents or accidents related to drug administration and their properties. These account for about 30% of all hospital adverse events (Henry et al. 2020). Such events can occur throughout the drug management process.^1^ Risks are associated with drug use. Therapeutic errors can cause toxicity or treatment failure.^2^ Medication errors may occur more often than system errors.^3^ Identifying and preventing medication errors, and promoting a safety culture, are essential for patient safety. Medication errors can be classified into six types: prescription errors, prescribing errors, transcription errors, dispensing errors, administration errors, and cross‐sectional errors.^4^ Each type can include wrong drug, dose, frequency, route, patient, or harm severity.^5^. Four main factors can influence medication errors: knowledge, attitudes, behaviors, and training needs. Inadequate knowledge and training in intravenous drug use among nurses can also lead to errors.^6^

## BACKGROUND

Chemotherapy is the main choice for treating or controlling cancer and is considered high risk by the National Institute of Occupational Safety and Health (NIOSH).^1^, ^7^ It involves using several adjuvant drugs aimed at a synergistic effect with minimal toxicity.^8^ The complexity of chemotherapy regimens and formulations increases the chance of clinical errors. Errors can occur during any step of antineoplastic drug management: procurement, storage, prescription, preparation, distribution, and administration.^9^, ^10^ Prescription and administration are especially risky.^11^, ^12^, ^13^ Chemotherapy administration is a vulnerable area in oncology nursing. Even minor negligence can negatively affect patients, staff, and the environment (Lee H. et al. 2021). Errors in administering multiple doses can be catastrophic or fatal.^14^ A US Poison Control report found chemotherapy drugs were often linked with medication errors. The most reported error was incorrect dosing (22.7%).^15^ German hospitals also reported incorrect doses most frequently.^16^

In this context, Failure Mode and Effect Analysis (FMEA) is a useful management tool to proactively identify potential errors and hidden risks in a system or process, analyze their causes and effects, and propose preventive measures to reduce the likelihood and severity of errors^17^, ^18^ and prevent possible defects in healthcare processes, often used to assess effects and risks on patients and analyze complex processes.^19^ In recent years, it has been widely and successfully used in different healthcare contexts, such as: healthcare process, hospital management, hospital computerization, equipment, medical manufacturing^15^ and healthcare of the chemotherapy preparation process to identify steps with the potential to cause errors and to develop further strategies to improve the process and minimize the risk of errors.^20^

This leads to understand that FMEA is a powerful risk analysis and risk management technique. The use of Failure Mode Effects and Criticality Analysis (FMECA) also allows for classification errors based on a Risk Priority Number (RPN). Recent studies on healthcare risk management highlight the role of nursing leadership and multidisciplinary governance in supporting proactive safety models over time.^21^, ^22^ These findings are consistent with the FMECA‐based approach, which integrates nursing competencies as a cornerstone for continuous safety improvement.

## THE STUDY

### Aim

The aim of this study is to conduct a prospective and systematic analysis of the critical phases that make up the chemotherapy process, applying the FMECA methodology to identify potential errors, prioritize subsequent interventions, and therefore allow the adoption of measures to prevent their occurrence.

### Objective

The general objective is to proactively analyze the process in order to identify high‐risk error modes and propose improvement interventions to increase patient safety.

### Research question

The research question is “What are the highest‐risk error modes in the management process of antineoplastic drugs, and which prevention strategies are effective in reducing them in our setting?”.

### Primary objectives

The primary objectives are: Identify potential error modes in the critical phases of antineoplastic drugs; Classify error modes according to frequency of occurrence, detectability, and severity, through the calculation of the RPN; Identify improvement actions to reduce the probability and impact of high‐risk errors; Evaluate the effectiveness of improvement actions by comparing the pre‐ and post‐intervention RPNs three months later.

### Secondary objectives

The secondary objectives are: Promote a culture of safety among healthcare personnel involved in the handling and administration of antineoplastic drugs; Provide a model of risk analysis that can be replicated in other oncology contexts.

## METHODS

### Design

A quality improvement study was conducted with a prospective and descriptive approach, using the FMECA methodology applied to the management process of antineoplastic drugs of oncological therapies at the Onco‐Hematological Department of the Scientific Institute for Hospitalization and Care—Oncology Reference Center of Basilicata (IRCCS CROB). The study was carried out between November 2024 and April 2025. The study was divided into five phases: Process mapping; Identification of potential error modalities; Classification of potential error modalities; Proposal and implementation of corrective actions; Evaluation of effectiveness. The study involved the proactive analysis of potential risks associated with the chemotherapy process, through the collaboration of a multidisciplinary team of experts with different experiences and technical skills^23^, which included production personnel who work on antineoplastic drugs or are part of the process.

The following criteria were established to select the team: having at least three years of experience in the chemotherapy sector, agreeing to participate in the FMECA development process, and being available to attend meetings. The multidisciplinary team consisted of risk managers, nursing coordinators, and oncology nurses from the units involved. Specifically, the team included: 1 clinical risk manager, 12 oncology nurses (including 2 nursing coordinators). Based on their area of clinical expertise, the oncology nurses were divided into two distinct groups of 6 nurses each: one belonging to the Antiblastic Chemotherapy Manipulation Unit (experts in the reconstitution, dilution and manipulation of chemotherapeutic drugs in a sterile environment), the other to the Oncology Day Hospital (experts in the intravenous administration of antineoplastic drugs and in the clinical management of the oncology patient).

### Study setting and data analysis

The work team was built as follows: the nursing clinical risk manager and an external FMECA expert observer, in order to specifically focus on the functioning pathway through the collection and creation of documents necessary for the analysis. Following these criteria, 6 expert nurses of the Antiblastic Chemotherapy Manipulation Unit and 6 expert nurses of the Oncology Day Hospital were selected. The second step dealt with a brainstorming process in which a list of possible risks and the most associated problems, linked to the aspects analyzed, was drawn up. At the end, the risks were examined individually. The next step involved the identification of all possible error modes and the search for the error causes divided by phase. Subsequently, for each error mode identified, the effects that it entails on the process were identified. The last step involved the risk assessment of the different error modes. Knowing that events do not all have the same probability of occurring, the same probability of being detected and not even the same severity. The evaluation scale used in previous FMECA studies on antitumor treatments^24^ was used, including the frequency of occurrence: probability that the event occurs, the detectability; probability that the error is discovered before it affects the patient, of the severity; the effect of the error on the patient (Table 1). Thus, the RPN was assigned, as usual in a FMECA study, multiplying the three factors: “occurrence x detection x severity”. The RPNs for the error modes in the preparation phase were assigned by the 6 expert nurses of the Antiblastic Chemotherapy Manipulation Unit, while the RPNs for the error modes in the administration phase were assigned by the 6 expert nurses of the Oncology Day Hospital. Each nurse individually rated the three factors, and then an average was calculated for each failure mode. The RPN allows to quantify and order the risk generated by the event in a quantitative/objective way, with the aim of establishing when an event requires urgent intervention or is acceptable to the point to postpone or delay the intervention. Knowing that the maximum RPN is 180, any RPNs>23 were chosen by the multidisciplinary group as a high‐risk error. This value corresponds to the 75th percentile and is used to establish the priority in error management. Errors with RPN>23, classified as high risk, were and identified as priority areas where it was necessary to implement safety strategies.

**TABLE 1:** Rating for frequency of occurrence, detectability and severity.

	Rating
*Frequency of occurrence of failure mode*	
Once a year	1
Once a month	2
Once a week	3
Once a day	4
Several times a day	5
*Detectability of failure mode*	
≥ 90%	1
≥ 75%	2
≥ 50%	3
≥ 25%	4
≥ 10%	5
0–9%	6
*Severity of the effect of the failure mode*	
Slight annoyance: may affect the cancer treatment system	1
Moderate system problem: may affect the patient	2
Major system problem: may affect the patient	3
Minor injury	4
Major injury	5
Terminal injury or death	6

### Ethical considerations

The study exclusively concerned the analysis of organizational processes and the implementation of quality improvement strategies. According to the regulations in force for this type of quality improvement studies, the approval of the Ethics Committee is not required. All the healthcare professionals involved participated on a voluntary basis and were adequately informed about the objectives and methods of conducting the study. The confidentiality of the data and anonymity during the collection, analysis, and presentation of the results were guaranteed. The study was conducted in compliance with the ethical principles stated in the Declaration of Helsinki and good clinical practices.

## RESULTS

The preparation and administration process of the antineoplastic drug was identified as a high‐risk activity to be analyzed. The analysis highlighted 8 possible error modes distributed in the phases of the preparation process (“Dilution and reconstitution”, “Contamination”, “Confusion between drugs”, “Inaccurate drug dosage”) and administration (“Failure to verify identity”, “Administration by incorrect route”, “Too rapid/slow infusion”, “Confusion or exchange of drugs”) of the antineoplastic drug with RPN ranging from 6 to 27 (Table 2). The sum of the criticality indices was 83 for the preparation phase and 36 for the administration phase. The error modes with the highest RPN were found in the preparation phase, among which were the error mode “Contamination” with RPN 27 and “Inaccurate drug dosage” with RPN 24, classified as high risk.

**TABLE 2:** Error modes in preparation and administration classified by risk priority number (RPN).

Error mode	Risk priority number
*Failure modes in preparation*	*83*
Dilution and reconstitution	12
Contamination	27
Confusion between drugs	20
Inaccurate drug dosage	24
*Administration error modes*	*36*
Failure to verify identity	6
Administration by incorrect route	8
Too fast/slow infusion	16
Confusion or exchange of drugs	6

Once the risks and the priority of the risks were established, it was time to identify and define the preventive and corrective actions in order to mitigate the risks. Therefore, for high‐risk errors, two prevention strategies were identified (Table 3). For the “Contamination” error mode, the recommended prevention actions were: Specific training on correct aseptic techniques and sterile manipulation; Use of partially closed‐circuit systems in place of open circuit systems. Both recommended prevention actions were implemented through: the training event entitled “Potential effects on the health of workers who handle mixtures of antineoplastic drugs” with the training objective of safety and hygiene in the environment and workplace and related pathologies; the use of partially closed‐circuit systems with partial hermetic closure and limited protection from contamination and aerosols, but safer than the open circuit system. For the error mode “Inaccurate drug dosage”, the recommended prevention actions were: Raising awareness and formalizing the double check of the drug dosage by the second operator (safety measure that provides for the verification of the correct drug by two operators) already in place but often not implemented; Use of calibrated measuring instruments for gravimetric preparation (analytical precision scales that allow drugs to be weighed precisely during their preparation following a gravimetric approach). Currently, one of the two recommended prevention actions has been implemented, the raising awareness and formalizing the double check by the second operator, through: updating the operating instructions, highlighting the activities carried out by the support nurse for the double check (supporting the preparing nurse in consulting the worksheet, in checking the preparation, and the residual volume of each bottle). The use of calibrated measuring instruments for gravimetric preparation has not been implemented due to consolidated habits, lack of binding guidelines, and the high workload, which pushes operators to prefer faster but less precise methods. After the identification of prevention actions, with the subsequent implementation, it was time for the measurement phase.

**TABLE 3:** Recommended actions to improve the preparation and administration phases of antineoplastic drugs.

Error mode	Recommended preventive actions
*Failure modes in preparation*	
Dilution and reconstitution	Use of automatic calculation software
Contamination	Specific training on correct aseptic techniques and sterile handling, Use of partially closed circuit systems
Confusion between drugs	Implementation of double check
Inaccurate drug dosage	Awareness and formalization of double control by the second operator, Use of calibrated measuring instruments
*Administration error modes*	
Failure to verify identity	Double ID verification
Administration by incorrect route	Clear and standardized labeling
Too fast/slow infusion	Use of infusion pumps with alarms
Confusion or exchange of drugs	Cross‐checking between healthcare workers

### Results after introducing corrective

After three months, the post RPNs (after the implementation of the improvement actions) were assigned for the error modes classified as high risk (Contamination and Inaccurate drug dosage) by the nurses of the Antiblastic Chemotherapy Manipulation Unit, who had already assessed the errors previously. For the overall evaluation of the results of the Analysis, a comparison of the initial RPNs (pre RPN) and those after the implementation of the improvement actions (post RPN) was carried out for each high‐risk error mode. Finally, the Improvement Index (II) was calculated (an II > 1 therefore indicates the reduction of the RPN after the improvement actions) as the ratio between the RPN pre/RPN post values (Table 4). After the implementation of the improvement actions, a reduction of the RPN of the error mode “Contamination” from 27 to 4 (−85.19%) and of the error mode “Inaccurate drug dosage” from 24 to 3 (−87.5%) was found. Respectively with an II of 6.75 and 8.

**TABLE 4:** Comparison between RPNs before and after implementation of improvement actions on high‐risk errors and improvement index (II).

Error mode	RPN pre	Improvement actions	RPN post	II
Contamination	27	Specific training on correct aseptic techniques and sterile handling, Use of partially closed circuit systems	4	6.75
Inaccurate drug dosage	24	Awareness and formalization of double control by the second operator	3	8

## DISCUSSION

The FMECA analysis allowed to identify, classify, and systematically address of the potential risks associated with the preparation and administration phases of antineoplastic drugs. The results obtained are particularly relevant as they provide concrete indications on the most critical areas of the chemotherapy process, even if each phase is potentially critical, offering ideas for improving safety and therapeutic efficacy.

### Identification of error modes and risk classification

The process led to the identification of 8 main error modes, divided between the preparation phase (4 errors) and the administration phase (4 errors). The most critical error modes, according to the RPN value, were found to be in the preparation phase: “Contamination”^25^, ^26^, ^27^ (RPN = 27) and “Inaccurate drug dosage”^28^ (RPN = 24). In accordance with the studies cited, both were classified as high risk in this study, exceeding the 75th percentile threshold (RPN > 23). These critical issues highlight how, in particular, the preparation phase^29^, ^30^ represents a delicate and risky moment, exposing both the patient and the nurses to potentially harmful events, similar to the administration phase widely described in the literature.^16^

### Improvement actions

The analysis provided practical recommendations for each error mode: implementation of automatic calculation software, training (delivered in a structured teaching program improves nurses’ knowledge and practice),^11^ closed‐loop systems (demonstrated reduction of surface contamination and ease of use),^31^ double checking (although it has limitations it is a reliable process),^32^ gravimetric preparation tools (detected dosing errors that would not have been detected with traditional methods),^33^ dual identification, standardized labeling, smart infusion pumps with alarms (have the potential to prevent 5% of administration errors)^34^ and cross‐checking. In particular, for the two high‐risk error modes: “Contamination” was addressed through training and the introduction of partially closed‐loop systems bringing the RPN from 27 to 4 (II = 6.75), while “Inaccurate drug dosage” saw the implementation of formalized double control, reducing the RPN from 24 to 3 (II = 8). These interventions have demonstrated a high positive impact on risk mitigation, highlighting the effectiveness of the combination of training, technology and structured procedures.

Although the analysis identified concrete and well‐structured improvement actions, in the case of the error “Inaccurate drug dosage” only one of the two preventive strategies was actually implemented, leaving a relevant aspect uncovered: the lack of calibrated measurement tools.^35^, ^36^ The reasons (lack of economic resources, consolidated habits and workload) highlight a systemic barrier that can compromise the long‐term effectiveness of prevention strategies. The measurement phase correctly highlighted a significant reduction in RPNs for high‐risk errors. However, the comparison between pre and post RPNs was carried out three months after the implementation of corrective actions, without a temporal follow‐up that could confirm the maintenance of the improvements over time. It would be useful to integrate the analysis with a longitudinal evaluation, to observe the real effectiveness in the long term and the possible need for readjustments. The effectiveness of the analysis will depend on the organization's ability to maintain the implemented actions over time, to fill the gaps that are still present and to promote a safety culture focused on continuous learning. An FMECA analysis, however detailed, is not a point of arrival, but a starting point for dynamic and constant improvement.

### Limitations of the work

The FMECA analysis conducted on the entire process of managing antineoplastic drugs represents an example of systematic application of risk assessment in the healthcare sector. However, while acknowledging its methodological merits and the effectiveness of the improvement actions adopted, some limitations emerge that deserve attention.

The use of the RPN indicator, calculated as the product of occurrence frequency, detectability and severity, strongly depends on subjective assessments of the individual nurses involved. This method, although involving expert nurses, can introduce variability in judgments with consequent significant differences in scores for the same error mode and reduce the reliability of the final score. Conservative experts may assign higher scores, while optimistic experts may assign lower scores. Differences in experience and knowledge among experts also influence the reliability of scores.^5^, ^10^, ^37^

Furthermore, the multiplicative method used to calculate the RPN assumes that the three factors have the same weight, which may not accurately reflect the real clinical impact of some failure modes. For example, a highly serious but infrequent event may have a similar RPN to a less serious but more frequent one, generating potential biases in intervention priority. Despite these limitations, the RPN represents and has represented a useful and immediate metric for pre‐ and post‐intervention comparison and for the definition of risk thresholds, supporting the orientation of corrective actions.^35^, ^38^

For future studies, it may be appropriate to use a flexible RPN or to combine the RPN with other risk assessment tools^39^, ^40^ or to explore more advanced and participatory weighting methods, such as the Delphi method.

Finally, it should be considered that the post‐RPN does not represent a direct measure of clinical or organizational outcomes. Therefore, its post‐intervention reduction indicates a perceived improvement in safety by healthcare workers, but does not guarantee a real decrease in errors. Future studies should combine the RPN with objective outcome or performance indicators to more directly measure the effectiveness of the implemented interventions.

### Recommendations for further research

Although this study has identified and reduced the risks associated with the process of preparation and administration of antineoplastic drugs through the application of the FMECA methodology, some areas emerge that require further investigation. A longer follow‐up is recommended to assess the stability and sustainability over the long term of the implemented improvement actions. It would be useful to replicate the analysis in other healthcare facilities and in different departments to verify the transferability and generalizability of the results. Future studies could explore the impact of active patient involvement strategies in preventing medication errors. Subsequent research should evaluate the impact of the introduction of gravimetric measurement tools and completely closed systems in drug preparation, which were not implemented in this study for organizational reasons.

Further studies could examine the organizational, cultural and economic barriers that may limit the adoption of recommended safety practices. These recommendations can help consolidate the effectiveness of interventions and promote an increasingly solid safety culture in oncology and healthcare settings in general. In addition to the recommendations proposed, two aspects deserve particular attention for the development of future projects: sustainability and generalizability.^36^, ^41^, ^42^ The sustainability of the improvement actions depends on their integration into the organizational structure and on the maintenance of continuous training, audit, and managerial support over time. Recent literature highlights that a strong safety culture and an active nursing leadership are essential elements to ensure the long‐term stability of safety practices.^21^ Embedding FMECA‐based processes within institutional policies, supported by regular re‐assessment of RPNs and continuous feedback loops, can help consolidate the gains achieved and maintain risk awareness across teams. Regarding generalizability, although the study was conducted in an oncological setting, its methodological approach is transferable to other high‐risk clinical contexts. Organizational health, defined by transparent communication, staff engagement, and psychological safety, strongly influences the effectiveness of safety interventions.^22^ Thus, replication of this FMECA model in diverse departments (e.g., intensive care, cardiology, emergency medicine) could help verify its adaptability and external validity. Future studies should therefore not only replicate this model in different healthcare contexts, but also measure its sustainability over time through longitudinal monitoring of clinical outcomes and staff perceptions of safety. These aspects would provide valuable evidence for scaling up proactive nursing‐led risk management strategies at the system level.

### Implications for clinical practice

The results obtained highlight how the FMECA methodology is a valuable tool for improving safety in oncology settings, where the complexity of treatments and the dangerousness of drugs make rigorous risk management essential. The adoption of standardized systems, the promotion of safety culture and the use of technological tools emerge as fundamental strategies.

## CONCLUSION

The adoption of the FMECA methodology applied to the management process of antineoplastic drugs has allowed us to systematically identify the main critical issues present in the preparation and administration phases. The analysis has highlighted how some error modes, in particular contamination and imprecise dosing of the prepared drug, represent high‐risk areas that require priority interventions. The effectiveness of the corrective actions implemented, measured through the significant reduction of RPNs, demonstrates the usefulness of the FMECA tool in guiding decisions in the field of clinical safety. However, for the improvements to be long‐lasting, it will be necessary to combine the continuous training of healthcare personnel with investments in technology, process standardization and an organizational culture oriented to prevention.

In conclusion, this study has laid the foundations for a risk management path in highly complex clinical‐care processes, which must be maintained and updated over time through regular monitoring and analysis of residual errors. Protecting patient and nurse safety depends not only on reactivity to errors, but on the organization's ability to proactively anticipate and mitigate them.

## AUTHOR CONTRIBUTION**S**

**Giuseppe Bertozzi, MD, PhD**: conceptualization (support); data curation (support); methodology (lead); supervision (equal credit); visualization (lead); writing—review and editing (lead). **Tommaso Cassano, MD, PhD, Full Professor**: project administration (support); supervision (equal credit); validation (lead). **Aldo Di Fazio, MD, PhD**: methodology (support); validation (support). **Gennaro Laus, Nurse Coordinator**: conceptualization (responsible); data curation (responsible); formal analysis (responsible); investigation (responsible); project administration (responsible); visualization (support); writing—preparation of original draft (responsible). **Federico Ruta**: data curation (support); formal analysis (support). **Sipontina Rita Zerulo, Nurse, Contract Professor**: conceptualization (support); methodology (support); project administration (support); supervision (equal credit).

## CONFLICTS OF INTEREST STATEMENT

The authors declare that they have no conflicts of interest to report.

## FUNDING STATEMENT

This study did not receive any specific funding from public, commercial, or not‐for‐profit funding agencies.

## ETHICS APPROVAL STATEMENT

According to current regulations, this quality improvement study, which only concerns the analysis of organizational processes and does not involve personal data or a clinical trial, did not require approval from the Ethics Committee.

## PATIENT CONSENT STATEMENT

It was not necessary to obtain patient consent, as the study did not involve individual clinical data or direct involvement of patients.

## IMPLICATIONS FOR THE PROFESSION AND/OR PATIENT CARE

This study highlights the importance of continuous training, the adoption of standardized procedures and the active involvement of nurses in the prevention of medication errors.

## REPORTING METHOD

Data were collected through direct observation, structured brainstorming and participatory evaluation by expert nurses.

## WHAT DOES THIS PAPER CONTRIBUTE TO THE WIDER GLOBAL CLINICAL COMMUNITY?

This document makes a concrete contribution to the global healthcare community presenting a systematic model of risk analysis applicable to the management of chemotherapy drugs, with a specific focus on the nursing role. The study provides evidence on the effectiveness of practical strategies to reduce high‐risk errors and proposes replicable interventions in other oncology settings. The methodology adopted can be extended to other high‐risk clinical areas, promoting a culture of safety based on prevention and continuous improvement of processes.

## ETHICAL CONSIDERATIONS

Ethical review and approval were waived because the investigation technique used is comparable to an observational study on the perception of healthcare workers. Informed consent was obtained from all subjects involved in the study.

## PERMISSION TO REPRODUCE MATERIAL FROM OTHER SOURCES

No copyrighted material from other sources was used.

## CLINICAL TRIAL REGISTRATION

The study is not a clinical trial and therefore has not been registered in a clinical trial database.

## Data Availability

The data supporting the findings of this study are available upon request from the corresponding author. The data are not publicly accessible as they involve internal organizational processes.
